# Co-creating Knowledge with Robots: System, Synthesis, and Symbiosis

**DOI:** 10.1007/s13132-022-00968-1

**Published:** 2022-02-14

**Authors:** Johanna Hautala, Jussi S. Jauhiainen

**Affiliations:** 1 School of Management, University of Vaasa, 65101 Vaasa, Finland; 2 Department of Geography and Geology, University of Turku, 20014 Turku, Finland; 3 Institute of Ecology and the Earth Sciences, University of Tartu, 50003 Tartu, Estonia

**Keywords:** Knowledge creation, Robot, Human–robot interaction, Spatial, Finland, Singapore

## Abstract

In the contemporary robotizing knowledge economy, robots take increasing responsibility for accomplishing knowledge-related tasks that so far have been in the human domain. This profoundly changes the knowledge-creation processes that are at the core of the knowledge economy. Knowledge creation is an interactive spatial process through which ideas are transformed into new and justified outcomes, such as novel knowledge and innovations. However, knowledge-creation processes have rarely been studied in the context of human–robot co-creation. In this article, we take the perspective of key actors who create the future of robotics, namely, robotics-related students and researchers. Their thoughts and actions construct the knowledge co-creation processes that emerge between humans and robots. We ask whether robots can have and create knowledge, what kind of knowledge, and what kind of spatialities connect to interactive human–robot knowledge-creation processes. The article’s empirical material consists of interviews with 34 robotics-related researchers and students at universities in Finland and Singapore as well as observations of human–robot interactions there. Robots and humans form top-down systems, interactive syntheses, and integrated symbioses in spatial knowledge co-creation processes. Most interviewees considered that robots can have knowledge. Some perceived robots as machines and passive agents with rational knowledge created in hierarchical systems. Others saw robots as active actors and learning co-workers having constructionist knowledge created in syntheses. Symbioses integrated humans and robots and allowed robots and human–robot cyborgs access to embodied knowledge.

## Introduction

Currently, robotization is sweeping globally through the knowledge economy, appearing in an increasing number of industries and across various cultures and places (Bauer, 2017; Bissell & Del Casino, 2017). Robots are both material and social, and shape society as people and society shape robots (Šabanović, 2010). Many robots are involved in standardized and repetitive mechanical actions. Some perform complex tasks independently and in interaction with humans in the constitutive entanglement of social and material in everyday lives (Orlikowski, 2007). A core example in the robotizing knowledge economy is knowledge-creation processes. There, ideas are transformed into new, valuable, justified, and trustworthy outcomes, such as novel knowledge and innovative products and services.

Today, various robots are involved in the knowledge-creation processes in varied spaces, which is why knowledge creation is no longer an exclusively human domain. There are many kinds of robots, including industrial arms; vacuum cleaners; autonomous vehicles and other mobile robots on land, air, and water; service robots; humanoids; soft robotics such as robotics in fabric; robotics (temporally) integrated with a human body like exoskeletons and bionic limbs; and swarms of small robot agents (Ricotti et al., 2017; Marasco et al., 2021). These robots exist in many environments. For long, robots have been present inside controlled spaces such as laboratories and factories, but now they exist also in the general population’s everyday environments (Anthes, 2017). Recent advances in cognitive robotics focus on improving robots with self-learning abilities, making robots’ learning procedural, higher in complexity, and more demanding than declarative knowledge (Zhang et al., 2021). New human–robot interactions are emerging and influencing knowledge co-creation processes (Meckin, 2019). Therefore, in the era of robotizing knowledge economy, the anthropocentric understanding of knowledge needs to be elaborated further.

In this article, we contribute to three specific research needs in the context of robots and knowledge-creation. First, there is a need to understand the changing knowledge co-creation processes in a robotizing knowledge economy (Carayannis et al., 2021). Very limited research exists thus far that combines robots and knowledge creation (excluding Lin et al., 2013; Hautala, 2021). To respond to this need, we take the perspective of the next generation of robotics engineers: robotics’ students and researchers creating the future of robotics. They develop robots, co-create knowledge with robots, and accomplish this with understandings of knowledge and relations between humans, robots, and knowledge. Therefore, robotics students and researchers are among the key actors in constructing the robotizing knowledge economy. To grasp such constructions, we analyze knowledge-creation processes at the micro-level of individuals in the next generation of robotics. This has been identified important in advancing the theory of knowledge creation (Bolade & Sindakis, 2020).

Second, to understand the emerging human–robot knowledge co-creation processes requires both conceptual and empirical research. In this article, we elaborate what a knowledgeable robot is (or could be) and focus on different types of human–robot interactions and related spatial aspects in knowledge-creation processes. According to the International Organization for Standardization (ISO 2.6, 8373, 2012), a robot is an “actuated mechanism programmable in two or more axes with a degree of autonomy, moving within its environment, to perform intended tasks.” We let the robotics’ students and researchers describe what they consider as robots. Most conceptualize robots as physically embodied, but a few accept robots also as digital autonomous systems such as chatbots (see also Blut et al., 2021; Fox & Gambino, 2021).

Third, there is a need to understand robots’ agency as well as the changing agency of humans who work with robots (Rose, 2017), in particular regarding knowledge-creation processes. Empirical research is needed on what a robot does in conjunction with humans in knowledge-creation processes. As mentioned, by their definition, robots are spatial actors (Hayles, 2017) moving within the environment and manipulating it in interactions with people. Therefore, understanding the connections among humans, robots, and space is critical (Lynch & Del Casino, 2020). There is a need to study human–robot interaction in the spaces of work in which knowledge and robots are developed simultaneously (Bryson, 2019; Del Casino, 2016; Del Casino et al., 2020; Lynch & Del Casino, 2020).

To answer these needs, we apply spatial and processual approaches to knowledge creation (e.g., Ibert et al., 2015). We consider robots as spatial actors creating knowledge through various human–robot interactions. This knowledge creation takes place in both controlled code/spaces of factories of which existence derives from a code as well as in more flexible places in which a code may be important but not fundamental (Kitchin & Dodge, 2011).

The research questions are as follows: (a) Can robots possess knowledge, and if so, what kind? (b) What are the relationships that humans and robots share in knowledge creation? (c) How does spatiality connect to the knowledge co-creation processes between robots and humans? The results are drawn from gathering and analyzing 34 interviews with robotics students and researchers as well as observations of human–robot interactions in lectures, seminars, and laboratory robot construction in one university in Finland and two universities in Singapore. Both countries are highly developed technologically and have advanced expertise in robotics. Thematic qualitative content analysis was applied to the material. Questions (a) and (b) are first answered conceptually by elaborating on various perspectives of knowledge, and second, empirically by analyzing human–robot interaction in knowledge-creation processes, including ways in which robots’ human developers discuss robots. Question (c) elaborates on the knowledge-creation theory further by analyzing spatiality’s role in knowledge co-creation processes between robots and humans. The article advances the current understanding of knowledge creation by introducing robots in it and the spatial dimension in human–robot knowledge co-creation.

## Robots: From Passive Agents to Active Actors in Knowledge-creation Processes

Humans and robots are actors in knowledge-creation processes. To elaborate the role of a robot, we apply Hayles’s (2017, pp. 31–32) differentiation among nonhumans. Agents or non-cognizers are material forces and artifacts (e.g., pens) that cannot act independently toward goals, although they might be needed in knowledge-creation processes such as writing ideas down on paper. Instead, actors or cognizers can make decisions. They include animals and devices (e.g., robots) that can act autonomously toward a goal. However, robots are fragile actors depending on electricity and batteries, and can be turned off and withdrawn from an agent position.

To discuss the role of robots in knowledge-creation processes, first knowledge must be defined, then whether and how a robot can have knowledge can be considered, and finally the role of robots in knowledge-creation processes can be explored. In terms of knowledge, robots’ agency is quite limited compared to that of humans. Knowledge is formed by tacit and explicit dimensions as well as of subjective and objective elements (Dodd et al., 2005; Sanzogni et al., 2017). Humans are active actors who “know more than we can tell” (Polanyi, 1983, p. 4). This refers to tacit elements of knowledge that are contextual, connected to individual experiences, values, and beliefs. Such elements of knowledge are subjective: a person is self-aware of their knowledge (Dodd et al., 2005). These elements in knowledge are embodied in a person’s practices as well as in cognitive and emotional processes. In fact, all human knowledge is connected to tacit knowing that can be embodied in the individual (somatic), located in a community (collective), or located in interactions (relational; Collins, 2010). Codified and objective elements of knowledge—messages, models, programs, and texts—can exist “outside” humans (Dodd et al., 2005; Nonaka & Takeuchi, 1995); thus, these are accessible for robots (Li et al., 2016). Robots can access explicit dimensions of knowledge, which can be beneficial in simple tasks. For example, when parking a car in a small space, a robot does not get nervous or think about previous experiences (Li et al., 2016). Tacit and explicit elements blend into a continuum and entity of knowledge through associations, argumentation chains, bodily practices, and experiences—“so that it is impossible to use one without the other” (Sanzogni et al., 2017, p. 38).

From a conceptual viewpoint, knowledge can be defined from rational, constructionist, and posthuman perspectives. Sanzogni et al. (2017) apply similar perspectives of knowledge to developing artificial intelligence. From the *rational perspective*, knowledge is “simple, certain, constructed by authority” (Muis et al., 2018, p. 167), objectivistic (Sanzogni et al., 2017), and empirically observable and measurable. It is extractable from humans into explicit codes, (mathematical) models, and databases (Forsythe, 1993; Hautala & Höyssä, 2017). Here, problems are identified and solved; thus knowledge is generated. Humans can distribute such explicit and objective knowledge elements and memory to devices, such as robots (Breazeal & Scassellati, 2002, p. 843; Li et al., 2016; Rose, 2017). In knowledge creation, human–robot interaction is hierarchical: humans control the robot and can reprogram it (Goodrich & Schultz, 2008, p. 2010; Sheridan, 2016) based on codified knowledge but robots can also assist humans in creating knowledge.

From the *constructionist perspective* (e.g., Knorr Cetina, 1999; Wenger, 1999), knowledge is “complex, tentative, actively constructed, and critically evaluated” (Muis et al., 2018, p. 167). Here, knowledge is based on subjective interpretation (Dodd et al., 2005; Sanzogni et al., 2017), and exists in people and their practices, networks, interactions, and epistemic cultures. Thus, humans and robots can form interactive collaborative teams (Azhar & Sklar, 2017). Information is exchanged from human to robot and from robot to human as indicated in “dynamic interaction” in human–nonhuman collaborative teams (Goodrich & Schultz, 2008, pp. 210, 231). This also considers tacit elements of knowledge creation.

In the *post-* and *transhumanist perspective*, knowledge is embodied and created in integrated humans–nonhumans and their practices (Hayles, 2017; Sanzogni et al., 2017; Watson & Huntington, 2008; Wolfe, 2010), for example, in actor networks (Latour, 2005). Technology is increasingly integrated into the human body unifying human and robot into a cyborg. One of the field’s key scholars, Rodney Brooks (2008), claimed he is a robot:

“while we become more robotic, our robots will become more biological, with parts made of artificial and yet organic materials. In the future, we might share some parts with our robots. A collection of technologies will emerge, mature, and enter our environments and bodies.”

Such a cyborg learns and knows as a cyborg (Haraway, 2006). A robot embodied in the human body becomes part of the human experience of tacit and subjective elements of knowledge. Here, intelligence emerges and operates within and across human–nonhuman relations and networks. Hayles (2017) calls this “neurodiversity,” indicating how knowing is entangled with cyborgs’ embodied cognition and consciousness that stretch beyond purely human and nonhuman. This perspective accepts that intelligence is a nonbinary process of knowledge production and consumption (Lynch & Del Casino, 2020).

The above-mentioned tripartite knowledge needs to be empirically elaborated in the current emerging robotics contexts. On the one hand, some scholars argue robots are agents without consciousness, thus they cannot know as humans do (Hayles, 2017), or enter directly an emotional and creative dialogue with a human (Jones, 2017). However, robots may enter such dialogue indirectly via human imagination: People may imagine robots as conscious and emotional beings (Jones, 2016, p. 7). For instance, people can express feelings such as love and trust toward robots (Turkle, 2006). In particular, the anthropomorphic appearance or behavior (e.g., human-like face, body, mobility, or using language) of social robots may prompt human users to treat them in human-like ways (Fox & Gambino, 2021). The ways in which humans can consider robots as coworkers can provide relevant knowledge in the knowledge-creation processes.

On the other hand, various scholars involved in robotics consider robots as active actors that can know. Engineering scholarship commonly states that the “robot knows,” but often without elaborating in depth upon what this knowing is. A robot’s knowledge is spatial: connected to environment, location, and mobility. Robots know about their physical, measurable environment through their information-collecting sensors (Rusu et al., 2009). To move properly to the intended destination, robots need to know locations (Cruz et al., 2019), directions, and speed (Das et al., 2007). Robots may need to communicate between each other or access each other’s information (Miao et al., 2018) to accomplish their tasks. Furthermore, robots can recognize humans and other robots’ gestures and facial expressions, and thus “know” about humans’ emotional messages and react accordingly to them (Luo et al., 2015, p. 535). Through such a “knowledge base,” robots can convince people to make particular decisions (Cheng et al., 2017, p. 336). This understanding of a robot’s knowledge is rational, considering knowledge as merely necessary information to achieve a goal.

A common approach among engineers has been to develop artificial intelligence by extracting from humans’ explicit elements of knowledge into codes. This codified and objective knowledge is then transferred to machines that use it for expected repeatable outcomes (Forsythe, 1993; Li et al., 2016; Carayannis et al., 2021). In knowledge-creation processes, tacit elements of knowledge are “sticky” and difficult to share between people (Bathelt et al., 2004). Sometimes such sharing is possible through long-term face-to-face interaction, learning-by-doing, and socialization into a community (Collins, 2001; Nonaka & Takeuchi, 1995). Even though general human–human relationship theories cannot be applied directly to examine contemporary human–robot relationships (Fox & Gambino, 2021), the roles and possibilities of robots in knowledge-creation processes are limited when considering knowledge as the continuum of tacit and explicit, or subjective and objective elements. However, robots are developed to overcome these limitations or enter knowledge-creation processes in other ways. For example, social robots can compensate for human shortcomings or even exceed human capacity (Fox & Gambino, 2021). Robots’ ability to read humans’ intentions creates trust and meaningful cooperation between humans and robots, which enhances the likelihood of a positive task outcome (Vinanzi et al., 2021).

To include robots in the study of knowledge creation requires seeing robots from the posthuman perspective that acknowledges robots as active actors. Following the actor-network theory, nonhuman objects (e.g., robots) can progress human thinking and allow, afford, or block human action (Latour, 2005, p. 72); can provide empirical evidence; back up argumentation; respond through error reports; learn; and move (Ahn, 2016; Dewey, 1997; Jensen & Blok, 2013; Jones, 2017; Kubo, 2013; Sele & Grand, 2016). A passive robot agent temporally can become an active actor in the knowledge-creation process if a human *decides* to interact with it. More advanced robots have “intentionality and potentionality” (Ash, 2018, p. 15), thus, such robots can become actors even if a human *does not decide* to initiate interaction (Hannibal, 2016; Skågeby, 2018). These robots include programs that apply machine learning, deep neural networks, and other forms of artificial intelligence that enable them to learn and enhance their actions on the go (Wu et al., 2013). Such reactivity allows robots, such as service robots and industrial arms, to work side-by-side with humans and to communicate with humans. This is done through movement, written codes, spoken language, facial expressions (Luo et al., 2015), imitation, surprises, and uncertainty—elements commonly referred to as “social learning” (Breazeal & Scassellati, 2002, pp. 484 − 485).

### Processual and Spatial Perspective on Knowledge Creation

In the knowledge-creation processes, robots differ from humans and simple objects. For humans, learning “is a part of all activity” and humans flexibly adjust their knowledge to different situations (Thomaz & Breazeal, 2008, p. 93). Robots lack such contextual flexibility, and without consciousness, robots are not able to know as humans do (Hayles, 2017). Even when using the best deep neural networks, robots “are locked into particular input and goal patterns” (Lake et al., 2017, p. 9). Enhancing the development of robots reduces such gaps. For example, robots are currently developed to recognize, simulate, react to, and extend humans’ emotions (Yan et al., 2021).

This article adopts a spatial processual perspective on knowledge creation when considering human–robot relations. Accordingly, knowledge is a *process* toward justified, interpreted, new, trustworthy, and valuable outcomes in the context in which knowledge’s novelty and relevance appear. The outcomes can be of many kinds: a peer-reviewed scientific article about robots, a new tool for a robot such as a hand, or a robot successfully completing a new task. Knowledge as a process is always becoming, tested, contested, and temporal—at times intensive, and at times on hold (Ibert et al., 2015; Langley et al., 2013).

Knowledge-creation processes are inherently *spatial* as the space that evolves in these processes shapes them*.* Spatial actors (mobile humans and nonhumans, e.g., robots) create knowledge in particular places (Livingstone, 2003). Current robotics research has concentrated on controlled spaces, such as laboratories, factories, and production lines (Hannibal, 2016; Tiddi et al., 2019). These spaces have various rules, regulations, and restrictions to ensure robots’ functionality, safety, and progress. However, space is not often considered in robotics research (Kitchin & Dodge, 2011, p. 13; Ash et al., 2018) despite robots are spatial actors by definition (ISO 2.6, 8373; 2012). They move in an environment while performing intended tasks. Even digital robots have an environment—a digital one. Many robots’ activities are about spatial cognition based on collecting, organizing, and analyzing data about an environment and interpreting this information within the contexts that connect it with meaning (Hayles, 2017; Lynch & Del Casino, 2020). Robots combine software as their brains and hardware as their bodies in material and digital spatial dimensions (Del Casino et al., 2020, p. 607).

The spatiality of human–robot knowledge-creation processes can be conceptualized by the terms “code/space” and “coded spaces” (Kitchin & Dodge, 2011). Robots and humans create *code/space* where “the software and the spatiality of everyday life become […] produced through one another” (Kitchin & Dodge, 2011, p. 16; see also Pink & Fors, 2017, p. 221). Here, space is produced through the written code woven into sociospatial relations and practices between humans and robots. For example, a robotized automotive factory is organized spatially and temporally through material and digital interactions to build a car step-by-step in the interaction between robots and humans. Such space emerges when the code is implemented. There, robots have clearly defined functional roles aimed at keeping unexpected events and actors outside. If this code fails, robots stop working, the functional factory (as a code/space) ceases, and eventually also this space. Therefore, the code/space and the environment in which robots act are often controlled spaces with well-organized hierarchical knowledge-creation processes.

In *coded spaces*, code is used to produce that space; however, that space’s functioning and existence does not depend on this code. Code facilitates that space’s functionality or efficiency; however, that code can be conveyed temporally without ceasing spatial functions (Kitchin & Dodge, 2011). For example, aiming to enter a restaurant, the staff can check physically the person’s digital COVID-19 certificate and identification card even if the digital device for it ceases to function. Therefore, even if the code designed for this space disappears, this functional space remains, even if in an altered form. Considering the knowledge co-creation process between humans and robots, the moments of interaction require interfaces to distribute meaning between humans, software, and hardware (Rose, 2017). Such moments create space through code/spaces (i.e., a code in a digital space is significant for a material space’s emergence). However, because humans are not dependent on robots (and codes) in the knowledge-creation processes, a shift from code/space to coded space does not prevent people from carrying on knowledge-creation processes.

## Material and Methods

This empirical study was conducted in Finland and Singapore. In Finland, the fieldwork took place at the University of Tampere from September 2018 to January 2019, and in Singapore at two universities from May to July 2019. All of these universities were advanced in robotics as academic fields. The University of Tampere was the first in the country to launch a major program in robotics, in 2017. In some Singaporean universities, robotics is a major subject, and in others, it is incorporated with other (engineering) subjects.

The empirical materials included thematic interviews with eight researchers and 26 students in robotics, observations of human–robot interactions, and a field diary the first author kept. The research received ethical approval by the university Ethics Committee in Finland (Statement 2/2019). All of the interviewees were contacted via an e-mail with information attached about the research and a privacy notice. The interviews were organized only with the participants who were willing to participate, and all of the participants were given the opportunity to read and comment on the manuscript before its submission for publication.

Each interview took about 30 − 60 min. The topics included human–robot interactions and knowledge dimensions related to robots. Two important notions were considered throughout the analysis (Table 1). First, the interviewees in Tampere included proportionally more students (84%) than in Singapore (67%), where students were more difficult to contact due to the visit occurring in summer. Therefore, the interviewees in Singapore were more experienced in working with robots. Second, only three women were interviewed. Robotics is generally a field that men dominate (Shi, 2018), which was also the case in the studied universities.

**Table 1:** Interviewees in Finland and Singapore

	Interviewees	Academic levels	Gender
Tampere	19	Student 16 Researcher 3	Men 17 Women 2
Singapore	15	Student 10 Researcher 5	Men 14 Women 1

In addition to the interviews, the first author visited the University of Tampere six times for 11 total days to observe two robotics courses (September 2018 − January 2019). The author participated in lectures, demonstrations, and seminars and observed students working in two robotics labs. In the first course, students formed project teams to design, build, and program a robot to deliver a task. They enhanced or combined existing robots in the lab. The students worked both independently and with the help of supervisors in the lab. The class frequently gathered with the teachers to discuss their progress and challenges. The second course combined lectures and lab work to program robots in teams. In Singapore, the first author received a tour of two robotics laboratories.

The interviews were held in English or Finnish and transcribed verbatim into text for analysis through qualitative content analysis (Hsieh & Shannon, 2005). According to Krippendorff (2018, p. 1), content analysis is an “empirically grounded method, exploratory in process, and predictive or inferential in intent*.*” The analysis included three stages. First, the relevant themes were identified and summarized in a table (rows: interviewees; columns: research questions, e.g., “What is a robot like?”). Second, new columns were added to summarize the content as key concepts (e.g., robots are material) that were used to construct the general categories (e.g., robots as only machines). Third, the diary was applied to compare the observation and interview findings and elaborate on the reasons for similarities and dissimilarities.

## Results

### Robots as only Machines and Learning Co-workers

In general, the majority of the interviewees considered robots as physical objects, and most did not consider the digital robots that were without a physical body to be robots. This might have been due to the interviewees’ experience of working mainly with physical industrial robots, which was especially the case among the Finland-based students. According to two-thirds (21/34) of all interviewees, a robot can possess knowledge. Eight participants believed that robots could not possess knowledge, while five referred to knowledge very generally.

When looking at the interviewees’ views on robots and robots’ abilities to possess knowledge more closely, two perspectives are visible. First is to see robots as only (stupid) machines that can possess knowledge from the rationalist perspective (16 interviewees). In this view, robots were defined as material devices that manipulate tangible objects and move in physical spaces. A robot was “a stupid device created by human beings” (Student 16/Tampere) and a “separate thing from a nonphysical artificial intelligence since it ‘must’ exist” (Student 17/Tampere). Robots have sensors (they can collect data about the physical space), control systems (they can analyze data through their software), and move physically as one of their outcomes:

[Robots are a] combination of motors, sensors, and code that is based on the data they produce. That [code] creates physical movement as the final outcome. (Student 4/Tampere)

[An] electronic device […] can move […][it has] some sensors and actuators. It need[s] to have something to take information and it need[s] to be able to do something with that information. (Student 8/Tampere)

In this group, 10 (of 16) interviewees believed that robots can have knowledge. Four thought that robots could not know “like we do” (Student 15/Singapore) because their “engineering is not that developed yet” (Student 14/Singapore). Robots cannot justify their knowledge or “explain why [their chosen] action is the best” (Researcher 2/Tampere). Such an understanding of robots’ knowledge (and its lack) is rational and similar to the view among artificial intelligence engineers in the 1990s (Forsythe, 1993). The knowledge robots hold is objective, explicit, possible to code, repeatable, domain specific, and bounded in a database and concerns the material-physical space:

Like what is the environment […] a human can know the alphabets, or know that this thing in here is a cupboard, […] [a] robot can know these kinds of things, too. (Student 6/Tampere)

If the robot has been taught that pike is a fish, it knows that a pike is a fish. (Student 17/Tampere)

Information experience about their environment […] will happen if A, B, C, and D are true. (Researcher 2/Singapore)

From this perspective, the human–robot relationship was hierarchical and unidirectional: humans control robots and their knowledge. Either a human collects information, creates a database, and transfers this to a robot, or the robot collects and analyzes the database on its own, but through code that a human has designed. A human sets the boundaries for the robot’s knowledge: “it has knowledge to the extent of […] what one [human] has defined it can have, not more than that” (Student 4/Tampere). Furthermore, knowledge was seen as separate, extractable, and transferable from the humans’ and robots’ bodies.

The second perspective was to consider the robots from a more collaborative view: as active actors or co-workers that could learn (17 interviewees). Knowledge in this group was considered from constructive perspective. Robots often were defined in relation to humans via comparison or co-evolution. These interviewees recognized robots and humans as becoming more like each other: “Robotics is understanding how we (humans) perform, behave, and think” (Student 15/Tampere). Robots’ (as only machines) simple materiality and physical motion were contested, and a few students accepted also purely digital robots as robots (Student 5/Tampere; Students 5, 10, 11, 13/Singapore, Researcher 14/Singapore). Robot’s digital immaterial “brains” were considered as an entity that could be (temporally) inserted into a material form: “We can treat them all as robots […] the computer is a robot. If we just grab electronic arms and legs and put them together, it's a robot” (Student 10/Singapore). The machines’ “brains” could form an actor network and collectively act upon the physical space (Ahn, 2016):

To be a robotic system, it doesn't really need to have motion per se, but it needs to *do* something. And that something doesn't have to be a physical motion. It can be when you enter a building, it's dark, then the lights turn on by [themselves]. (Researcher 4/Singapore)

In this group, 11 (of 17) interviewees thought that robots could have knowledge. Four thought they could not for similar reasons as those who considered robots as only machines. One aspect consisted of the explicit elements of knowledge, here in comparison to humans:

We represent the process through mathematics. […] robots in artificial neural networks [...] [interpret] what’s going on over many data sets. So, this is possibly the closest parallel […] between robot knowledge and human knowledge. (Student 6/Singapore)

The interviewees who viewed robots as learning co-workers emphasized knowledge from a constructionist and processual perspective, which consisted of learning, understanding, making sense, and reasoning. This was generally related to tacit and subjective elements of knowledge:

Learned, observed, and shared: Knowledge can be used for reasoning and decision-making or to extract new information. (Researcher 1/Tampere)

Knowledge is understanding […] how things work, […] It doesn’t mean truth. It’s a way to describe, but there may be alternative ways to describe it. (Researcher 14/Singapore)

Those who considered robots as only machines described robots’ ability to have knowledge *about* the physical space, whereas the robot as a learning co-worker learned to become an autonomous actor *in* a physical or digital space. The former referred to coded space through using code to give that space function. The latter referred to code/space in which space emerged through the robots’ function. Such autonomy required robots to learn, interact with their environment, and react on the go: “even [if] a new event or new decision is coming, he can take this on his own. Based on previous experience, he has gathered on this working’” (Student 11/Tampere). Thus, such robots could form collaborative spatial practices with humans in knowledge-creation processes.

Although these interviewees considered robots as learning co-workers, they saw a hierarchy between humans and robots because humans created the programs that allowed the robots to learn. However, robots sometimes initiated communication with humans and brought them uncertainty and surprises. Such a robot “should do something that you don’t tell it to do’” (Student 5/Tampere), so that “you don’t know how the robot is going to behave” (Researcher 4/Singapore). Here, knowledge can be considered embodied in the practices of individual humans and robots and knowledge can move between the bodies of humans and robots.

### Spatial Knowledge Co-creation Processes: System, Synthesis, and Symbiosis

Human–robot interaction was connected to three knowledge co-creation processes with different dimensions of space—code/space, coded space, and redefined relational space—that emerged from relations among humans, robots, and cyborgs. Sometimes, the interviewees distinguished between digital and material, such as in programming (code) versus its actual realization (robots using the code). In other cases, material and digital were integrated into one space. Most of the interviewees discussed allocating work between humans and robots in terms of a system or synthesis, but they also provided examples of symbiosis (Table 2).

**Table 2:** Human–robot interaction in knowledge co-creation processes

	**System**	**Synthesis**	**Symbiosis**
Robot	Only machine	Learning co-worker	Cyborg
Human	Creative engineer	Learning co-worker	Cyborg
Interaction	Hierarchical	Co-creative	Integrated
Knowledge	Divided rational	Shared constructionist	Embodied
Knowledge-creation process	Extraction and transfer	Co-creation	Being
Space	Controlled code/space	Everyday communicative code/space and coded space	Redefined relational space

### Systems: Humans’ and Robots’ Separated Knowledge in Controlled Code/Spaces

In systems, robots are “only a machine” the creative engineer controls. Robots and humans have different and separated knowledge. In humans’ knowledge, the emphasis is on tacit and subjective elements needed to create “novelty,” justify knowledge, understand emotions and deep communication, and connect robots’ routine work to the knowledge-creation process. Human creativity, in general, was considered to exceed the robots’ agency (Rose, 2017, p. 782). Creating novel knowledge is based on its extraction (Forsythe, 1993) from experts’ brains. It is then logically compiled into truthful scientific documents, databases, and code (Kim & Lee, 2019). This is an information flow from humans to machines (Breazeal & Scassellati, 2002, p. 483) and extended memory (Rose, 2017) transferred (uploaded) to a robot, which then can perform repeatable tasks humans designed. Such knowledge is not dynamic but has predictable outcomes (Lee & Helgesson, 2019). Thus, robots participated in the knowledge-creation processes operationally with their accurate (i.e., trustworthy), ongoing, and routine repeatable work.

Robots can do work that is repeatable, programmable, [and] easy, [but] not funny. (Student 7/Singapore)

Like the surgery robots, they only repeat the moves of the human as accurately as possible. (Student 4/Tampere)

The knowledge-creation process has been transformed to meet a controlled code/space. As a result, both work and workspaces have been reorganized into a sequential order of stages, production lines, and specific activities (Holloway, 2007). Extracting knowledge into separate tasks and creating new combined knowledge require a high degree of control and keeping unexpected happenings outside. For example, in Tampere, the robotics students felt that knowledge had been created when robots delivered the task exactly as the students aimed. Their code produced the robot’s visible movement. This merged digital with material and created a code/space. In another case, the students programmed a robot to move an object from one place to another, which it did. However, the instructor saw that the robot’s gripper had closed while its hand was still moving, but the students disagreed because they had not seen this. The instructor found that the code command was on the wrong line, and only then did the students understand the mistake in the code and the robot’s movement. This resembles rational knowledge: The code *must* produce the right outcome that humans observe and what *must* be understandable through the code. If these were not true, the robot could not be trusted to repeat the activity. Human–robot systems require engineer coding experts.

When the code does not work, the process is suspended. Rose (2016) would call these frictions of human–software–hardware interfaces. On various occasions, the code worked perfectly in the digital simulation, but the robot’s material action did not work properly. The perfectly designed code of digital space met, in physical space, wires in the wrong places, sensors that did not recognize glass walls, grippers that did not grip specific objects, etc. These cases could halt the knowledge-creation process between humans and robots.

### Synthesis: Knowledge Co-creation Between Humans and Robots in Communicative Code/Spaces

I’d say that if a robot is working together with a human to solve a problem, then the system [here: synthesis] as a whole has knowledge. But maybe the robot itself doesn’t have the knowledge; maybe the human cannot do it on its own. (Student 8/Singapore)

In synthesis, humans and robots form connected units of actors that can create knowledge *together*. Such learning co-workers include cognitive assemblage (Hayles, 2017) and social machines as “a single problem-solving entity” (Minimair, 2018, p. 194). The interviewees presented synthesis as an ideal that required more advanced robots, but also saw that the first steps had been taken. In comparison to the human–robot systems, the knowledge elements are not divided and separated between humans (tacit elements) and robots (explicit elements). Interviewees acknowledged that not all knowledge “comes down to numbers” (Student 6/Singapore), similar to the skill-based essence of painting. Thus, in synthesis, humans should also share tacit knowledge elements with robots, for instance, in the form of embodied practices.

Human–robot collaboration is also a very unknown thing to me, because how does a robot ask for help? How do you tell the robot what you want it to do? […] maybe you show [it] how you cook, you show [it] how you do everyday things […] [An] ideal robot is something with basic capabilities already. (Researcher 5/Singapore)

Humans and robots are co-workers that learn from each other. This makes the hierarchy (mostly) disappear from their interaction, thus expanding the robots’ role in the knowledge co-creation process. In addition, people without coding expertise can create knowledge with robots. The emphasis changes from rational knowledge in code/space to merging digital and material in the human–robot interaction. The knowledge creation emphasizes the communicative dimension in code/space and coded space. Robots can participate in novel creation and justify and move knowledge from themselves to humans.

The work of the students who were building and programming robots included moments when the strict rules and control of the laboratory space were bypassed. Students played with robots, improvised and built grippers with shoelaces, combined robots to explore and extend their activities, and imitated their behavior to understand the reasons and gain suggestions for their unexpected actions. Robots’ activities triggered ideas about how to develop the project further. However, releasing the robots from controlled laboratories could have led to unexpected and risky outcomes. The ongoing ability to identify, interpret, and react to events requites robots’ constructionist knowledge, instead of the rational knowledge that prevails in human–robot systems.

Anything can go wrong, so can we really release them? […] In factories, […] things are deterministic, so we are used to [telling] robots and machines to be perfect, don´t do mistakes. When they come outside, things are dynamic, they are uncertain. […] No matter how perfect they are they will get into accidents. […] If somebody just jumps in the front of the car, [the] car has initial speed so it can't be stopped no matter how intelligent you are. (Researcher 3/Tampere)

### Symbiosis: Redefining Knowledge by Spatially Embodied Cyborgs

In symbiosis, robots become (temporally) part of humans, and humans and robots integrate into each other as cyborgs. Humans “are no longer contained—or even defined—by the boundaries of their skins” (Hayles, 2017, p. 2). An example of symbiosis was a wearable electromechanical device (exoskeleton) that assisted humans, such as to move limbs. Exoskeletons have been used, for example, in rehabilitating injured people so they can use their limbs or to reduce the strain on the body during the physical work in factories (Sylla et al., 2014). Several interviewees in Singapore had worked with wearable robotics and described deep communication and “co-being” between humans and robots:

The closer the interaction with humans, the more difficult it is. [...] [This is a] very high level on human–robot collaboration because you are literally touching the robot. (Student 6/Singapore)

The experience of using exoskeletons is a process of “embodiment” re-enabling the body and its abilities (Pazzaglia et al., 2013); that is, “The intelligence is embedded within a structure” (Student 6/Singapore), indicating the plasticity and cognition in the cyborgs’ body schema (Longo & Serino, 2012, p. 230). Thus, robots are inserted into a person’s body to overcome the human body’s limits and acquire new embodied knowledge by being (temporally) a cyborg (Abrahamsson & Simpson, 2011). However, the interviewees had very little, if any, experience of cyborgs’ fully integrated embodied knowledge. Such human–technology assemblages have been discussed as learning and as moving a “quantified self” (Lupton, 2016) and as “digital wayfarers” (Pink & Fors, 2017) in digital-material spaces. Merging with humans allows robots to access tacit elements of knowledge and practices, so that robots create and possess constructionist and embodied knowledge. In symbiosis, the robot co-evolves with humans (Lupton, 2017, p. 4). Cyborgs re-define space in practice through their novel movement and work— different from not only those of humans or robots, but also their co-working. Examples of re-defining spatialities and embodied knowing include cyborgs’ “superhuman senses” integrated with individual experience, cognition, and memory (Wheeler, 2018, p. 1).

## Discussion

The next generation of robot engineers—university robotics students and researchers—are at the forefront of creating the future of robotics, human–robot knowledge-creation processes, and the robotizing knowledge economy. They approach this with their perspectives on knowledge, on different understandings of robots as knowledgeable actors, and varied human–robot relationships in spatial knowledge-creation processes. We investigated these perspectives in this article through robotics university students and researchers (including their supervisors) in Finland and Singapore. This is one of the first studies to connect robots and knowledge creation, thus it is an important contribution to understand empirically and theoretically the changing knowledge and knowledge-creation processes in the robotizing knowledge economy currently being constructed (Sanzogni et al., 2017; Meckin, 2019; Bolade & Sindakis, 2020; Carayannis et al., 2021).

First, students and researchers identified three emerging relations (system, synthesis, and symbiosis) in which humans and robots had particular roles. In their visions, the human’s roles vary from that of top-down controller (systems) to mutual co-worker (synthesis) and integrated cyborg (symbiosis). In systems, humans are in control and perceived to pass to robots all necessary knowledge of mundane tasks. In synthesis, humans and robots collaborate and co-creatively learn from each other. In symbiosis, humans and robots become integrated and extend their skills beyond the limitations of pure humans and pure robots in the knowledge-creation processes. Moreover, each relation revealed different (rational, constructionist, and embodied) understandings of knowledge and different roles of humans and robots in spatial knowledge co-creation processes. Identifying these relations contributes to the call for better understanding not only robots’ agency, but also humans’ changing agency (Rose, 2017).

Second, the article revealed that the most common framework of current human–robot interactions among the interviewees was to understand knowledge as rational and hierarchical. In human–robot systems, humans (engineers) control robots. This is based on rational knowledge and has for long been common among artificial intelligence and robot developers (Forsythe, 1993). Here, strong, precise, and relentlessly working robots gain their knowledge from humans and have a justified place alongside humans in the controlled code/space of laboratories and factories. In such a human-based knowledge economy, the winners would be creative engineers who master coding and create new ideas and knowledge. However, critiques have been addressed to such hierarchical relationships in which knowledge narrowly flows only from humans to robots (Breazeal & Scassellati, 2002). Here, robots contribute to knowledge creation narrowly by only repeating specified human-designed activities with exactly foreseen outcomes, and only humans bring knowledge due to its novelty, value, and critical justification. Separating tacit and subjective (human) and explicit and objective (machine) knowledge neglects knowledge creation’s processual dimension. In all human–robot interactions, multiple meanings of knowledge are at work simultaneously.

However, the next generation of robotics engineers has started to apply also constructionist and embodied approaches to knowledge, and want to develop robots along these lines. Most of the interviewed robotics students and researchers considered that robots could have knowledge—if not independently, then in synthesis and symbiosis with humans. If robots could learn non-mathematical languages (e.g., speech), then not all robots’ human co-workers would need to speak the code’s language. However, “computers can never step outside the code, reflect on the code, and contribute their own observations” (Salzogni et al., 2017, p. 47). Thus, robots can access tacit elements of knowledge, embodied knowledge, and related social practices only through humans. As robotization advances, robots would be needed as companions (Lupton, 2017) that know *with* humans, forming synthesis and symbiosis. The currently prevailing anthropocentric understanding of knowledge becomes too narrow and must be elaborated further. Such knowledge would not be *post*-human: it would be more than human and created in various modes of human–robot interactions. Critical reflections between engineers and social scientists are needed to discover the possibilities of different perspectives on knowledge in developing robots and human–robot interactions. This article lays the groundwork for further research that is required to properly advance the theory of knowledge creation in the robotizing knowledge economy. Empirical research is needed to widen the context from university to industry, entrepreneurs, and the public sector, for instance, to employees and managers in robotized factories, surgeons in hospitals, artists, etc.

Third, the article brought a novel perspective to the spatiality of knowledge-creation processes (Hautala & Jauhianen, 2014) through human–robot interaction. Robots are spatial beings, and their activities connect material and digital dimensions of space (Del Casino et al., 2020). This activity is also referred to as spatial cognition: collecting, organizing, and analyzing the environment’s data (Hayles, 2017; Lynch & Del Casino, 2020). This study showed that controlled code/spaces support the efficient work of humans with robots. However, these spaces’ functionality is fragile and several frictions can stop the knowledge creation there. Empirical research in robotics is needed also outside the controlled spaces of laboratories, factories, and production lines (Hannibal, 2016; Tiddi et al., 2019). From the perspective of knowledge creation, the everyday spaces of human–robot interaction challenge the ways in which robotics mainly has been developed in higher education institutes. When top-down control and pre-set regulations were dissolved, the robotics researchers and students became more receptive to robots’ “suggestions.” This enhanced the knowledge-creation process. In a robotizing knowledge economy, it is important to deepen the connection between robotics scientists and social scientists to understand how more-than-human knowledge creation develops and what embodiments of knowledge emerge in the intensifying interactions and integration between humans and robots.
